# Pellicle formation in *Shewanella oneidensis*

**DOI:** 10.1186/1471-2180-10-291

**Published:** 2010-11-16

**Authors:** Yili Liang, Haichun Gao, Jingrong Chen, Yangyang Dong, Lin Wu, Zhili He, Xueduan Liu, Guanzhou Qiu, Jizhong Zhou

**Affiliations:** 1 School of Minerals processing and Bioengineering, Central south University, Changsha, 410083, PR China; 2 Institute for Environmental Genomics and Department of Botany and Microbiology, University of Oklahoma, Norman, 73019, USA; 3 Institute of Microbiology and College of Life Sciences, Zhejiang University, Hangzhou, Zhejiang 310058, PR China

## Abstract

**Background:**

Although solid surface-associated biofilm development of *S. oneidensis *has been extensively studied in recent years, pellicles formed at the air-liquid interface are largely overlooked. The goal of this work was to understand basic requirements and mechanism of pellicle formation in *S. oneidensis*.

**Results:**

We demonstrated that pellicle formation can be completed when oxygen and certain cations were present. Ca(II), Mn(II), Cu(II), and Zn(II) were essential for the process evidenced by fully rescuing pellicle formation of *S. oneidensis *from the EDTA treatment while Mg (II), Fe(II), and Fe(III) were much less effective. Proteins rather than DNA were crucial in pellicle formation and the major exopolysaccharides may be rich in mannose. Mutational analysis revealed that flagella were not required for pellicle formation but flagellum-less mutants delayed pellicle development substantially, likely due to reduced growth in static media. The analysis also demonstrated that AggA type I secretion system was essential in formation of pellicles but not of solid surface-associated biofilms in *S. oneidensis*.

**Conclusion:**

This systematic characterization of pellicle formation shed lights on our understanding of biofilm formation in *S. oneidensis *and indicated that the pellicle may serve as a good research model for studying bacterial communities.

## Background

Most microbes in natural ecosystems exist in highly organized and functional interactive communities, which are composed of cells attached to surfaces and/or to each other either from a single species or multiple species [1-7]. Microbial communities confer a number of advantages for survival, such as nutrient availability with metabolic cooperation, acquisition of new genetic traits, and protection from the environment [4,8]. The most common microbial communities are biofilms, which refer to assemblages of cell on solid biotic or abiotic surfaces. In recent years, the subject of microbial biofilms has drawn a lot of attention and numerous studies have provided important insights into the genetic basis of biofilm development [5,7].

Pellicles, arising from the interface between air and liquid and therefore frequently called air-liquid (A-L) biofilms [9], have been well studied in an array of bacteria, such as *Bacillus subtilis*, *Pseudomonas aeruginosa*, and *Vibrio parahaemolyticus *[7,10-12]. Pellicle formation consists of at least three distinctive steps: (i) initial attachment of bacteria to the solid surface (wall of culture device) at the interface between air and liquid, (ii) development of the monolayer pellicle initiated from the attached cells, and (iii) maturation of pellicles with characteristic three-dimensional architecture [1,11]. In addition to cells, a variety of components, mainly extracellular polymeric substances (EPS), are needed for developing and maintaining the pellicle matrix. The most extensively studied EPS include exopolysaccharides, proteins, and extracellular DNA although contributions of these agents to the integrity of the pellicle matrix may vary [11]. While the pellicle is generally taken into account as a special form of biofilms [5,7,13], its distinguishing characteristics justify that this type of biofilm may serve as an independent research model [12-14].

Many factors, including extracellular organelles such as flagella and type IV pili, secreted proteins, and chemical agents supplemented in media such as iron and phosphate, have been shown to play important roles in biofilm formation [5]. However, effects of these factors on the biofilm formation process depend on the bacterium under study. For example, flagella facilitate surface adhesion for many species but it has been also observed in other species that mutations resulting in aflagellate and paralyzed nonmotile cells promote formation of a multilayer biofilm [7]. In the case of iron, results are even more inconsistent. In *P. aeruginosa *and *Vibrio cholerae*, iron limitation hinders biofilm formation whereas it facilitates the process in *Actinomyces naeslundii *and *Staphylococcus epidermidis *[15,16]. It has been suggested that variation in effects of these factors on biofilm formation by particular species of bacteria may be reflection of the different environmental niches where they live [14,17-19].

*Shewanella oneidensis *MR-1, a facultative Gram-negative anaerobe with a remarkable respiratory versatility, has been extensively studied for its biofilm development [20-26]. However, little progress has been made to understand biological mechanisms of pellicle formation. This work represents the initial steps in characterizing the process in *S. oneidensis*. We showed that successful pellicle formation required the availability of oxygen and the presence of certain metal cations. A further analysis on metal cations revealed that Fe(II) and Fe(III) were not as essential as Ca(II), Cu(II), Mn(II), and Zn(II) for pellicle formation. In addition, results presented demonstrated that a type I secretion pathway of *S. oneidensis *is required for the pellicle development but not for attachment to abiotic surface.

## Results

### Characteristics of *S. oneidensis *growth in still media under aerobic conditions

**Figure 1:**
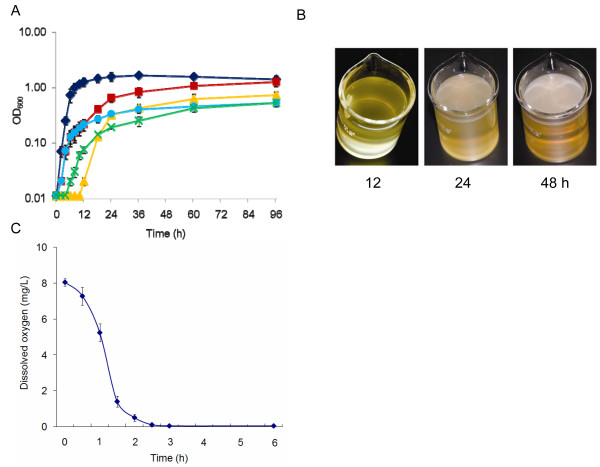
**Pellicle formation of *S. oneidensis *in LB under aerobic conditions**. (A) Growth of *S. oneidensis *in static liquid LB under aerobic conditions. Cell density of all cells (planktonic and pellicle cells combined) (brown square), pellicle cells (yellow triangle), planktonic cells (blue circle), and the Δ*flgA *mutant (green cross) was shown. Growth of agitated cultures (black diamond) is included for comparison. Presented are averages of four replicates with the standard deviation indicated by error bars. (B) Pellicle formation of MR-1 in static liquid LB under aerobic conditions. The pellicles started to form about 12 h after inoculation based on the altered growth rate of planktonic cells at the room temperature. (C) Dissolved oxygen concentrations at 1 cm below the surface in the static MR-1 cultures.

The *S. oneidensis *MR-1 cells grew rapidly in LB in a flask when aeration of the media was provided by vigorously shaking, with a doubling time of approximately 40 min at the room temperature (Figure 1A). Such growth eventually led to formation of the solid surface-associated (SSA) biofilms on the flask wall, especially around the A-L interface. Cells in static media accessible to ambient air, however, displayed a different growth pattern. Before pellicles were formed, cells lived in the planktonic form with a much longer doubling time, approximately 2.6 h (Figure 1A). Once pellicle formation initiated, some of the planktonic cells started to form pellicles while the rest remained in the planktonic form. During the development of pellicles, the planktonic cells grew at a much lower rate with a doubling time of approximately 6 h (Figure 1A). In this study, initiation of pellicle formation was determined by the time point where the growth rate of the planktonic cells changed although pellicles visible to naked eyes appeared much later, about 12 hours after inoculation at the room temperature. Both complex and defined media supported pellicle formation of *S. oneidensis*. However, pellicles from LB were thick and fairly uniform compared to thin and porous ones from the defined medium, indicating an impact of nutrition on pellicle formation (Figure 1B). We therefore chose LB through the rest of this study unless otherwise noted.

### Oxygen is required for pellicle formation in *S. oneidensis*

As demonstrated above, *S. oneidensis *initiated the pellicle formation process under aerobic conditions. We then asked whether oxygen is an essential factor for pellicle formation of this microorganism. The pellicle formation assay was carried out under anaerobic conditions with lactate as the electron donor and one of following agents as the electron acceptors: fumarate (20 mM), nitrate (5 mM), DMSO (20 mM), TMAO (20 mM), or ferrous citrate (10 mM). In all cases, the capacity of *S. oneidensis *cells to form pellicles was abolished (data not shown), indicating that oxygen is required for the process. This is in agreement with the findings that the lack of oxygen also resulted in a defect in SSA biofilm formation and a sudden decrease in oxygen concentration led to rapid detachment of SSA biofilms [25,27].

To further elucidate the role of oxygen in pellicle formation, dissolved oxygen concentrations (DOC) at four different depths below the surface in the unshaken cultures were measured in a time-course manner. Results revealed that DOC at 0.5, 1, and 2 cm below the surface in the unshaken cultures displayed a similar declining pattern with time, decreased rapidly from approximately 8 to 0.04 mg/L during the first two and half hours, and then remained stable at 0.04 mg/L (Figure 1C). However, DOC at the depth immediately below the surface (0.1 cm but the detector immersed in the liquid) reduced in a much slower rate and reached the lowest level of 0.04 mg/L only after the pellicle formed. These data indicate that the majority of dissolved oxygen is likely consumed by the cells close to the surface and the cells below the surface were grown under microaerobic/anaerobic conditions even before the pellicle was formed.

### Proteins are essential in pellicle formation of *S. oneidensis*

**Figure 2:**
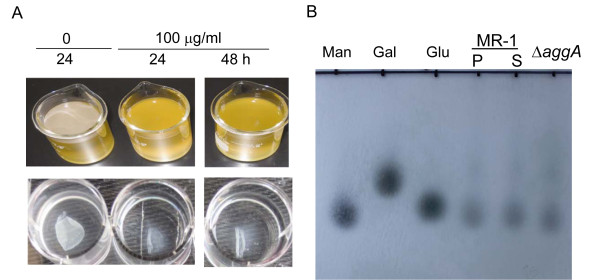
**EPS analysis**. (A) Effects of proteinase K on pellicle formation and developed pellicles. Upper-panel, pellicle formation of the WT in static LB, in which the proteinase K was added at inoculation to 100 mg/ml (final concentration). Lower panel, developed pellicles of the WT (48 h after inoculation) were treated with 100 mg/ml (final concentration). (B) TLC analysis of monosaccharide in pellicles and supernatants. P and S represent pellicle and supernatant, respectively. Man, gal, and glu represent mannose, galactose, and glucose, respectively. Supernatants of the *aggA *mutant culture were included in the analysis.

Since EPS, including proteins, polysaccharides, extracellular DNA, humic acid, and sugar, are important in SSA biofilm and pellicle formation of various bacteria, we speculated that these biopolymers may play a role in pellicle formation of *S. oneidensis*. To this end, effects of proteinase K and DNase I on pellicle formation and developed pellicles were assessed. The pellicles were prevented from formation in the presence of 100 μg/ml proteinase K (Figure 2A). Consistently, 100 μg/ml of the proteinase K was able to degrade the developed pellicles in 24 h, resulting in the semi-transparent membrane-like complexes (Figure 2A). In the control experiment, proteinase K at concentrations up to 300 μg/ml did not show a noticeable inhibitory influence on growth of *S. oneidensis *under agitated conditions. On the contrary, DNase I (up to 1000 U/ml) was not effective to inhibit pellicle formation or to degrade of the developed pellicles (data not shown), suggesting that DNA plays a negligible role in the process. Since proteinase K unspecifically removes polypeptides in the extracellular space and in the outer-membrane exposed to environments, the results could not conclude whether specific extracellular proteins are required for the process.

Attempts were made to solve the major polysaccharide components of *S. oneidensis *pellicles by the thin layer chromatography (TLC) analysis. Culture supernatants and pellicles were collected independently after 36 h of growth and pellicles were then treated with 100 μg/ml proteinase K to removed cells. Polysaccharides were extracted and subjected to TLC analysis as described in Methods. A preliminary experiment was performed with six monosaccharides as standards, including ribose, mannose, glucose, galactose, rhamnose, and N-acetyl-glucosamine. The monosaccharides visualized on the TLC plates were close to mannose, glucose, and galactose (data not shown). To further confirm the observation, the experiment was conducted again with these three monosaccharide standards only. As shown in Figure 2B the major monosaccharides identified were most likely to be mannose in both supernatants and pellicles. To validate this result, the *aggA *mutant, a pellicle-less strain was included in the analysis and the same result was obtained. These data suggest that the mannose-rich polysaccharides identified in pellicles are not pellicle specific.

### Certain metal cations are required for pellicle formation in *S. oneidensis*

**Figure 3:**
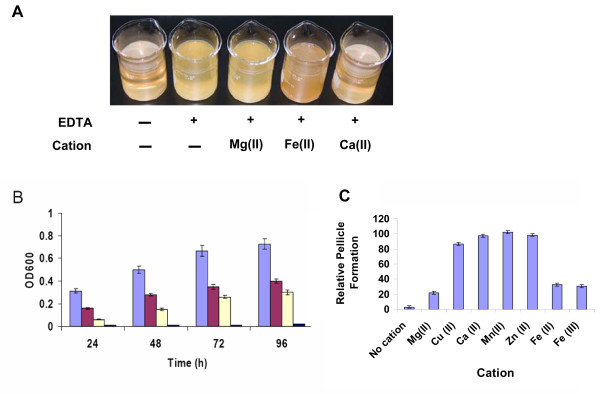
**Treatment of *S. oneidensis *pellicles with EDTA and divalent cations**. (A) Pellicle formation of the WT after 48 h in static LB in the presence of 0.3 mM EDTA and certain divalent cation (0.3 mM) under aerobic conditions. (B) Cells in pellicles formed in the presence of 0 (light blue), 0.1 (dark red), 0.2 (light yellow), and 0.3 mM (dark blue) EDTA at the different time points. Presented are averages of four replicates with the standard deviation indicated by error bars. (C) Effects of divalent cations on the inhibition of pellicle formation by EDTA. Pellicle formation of the WT after 48 h in static LB in the presence of 0.3 mM EDTA and one of indicated divalent cations (0.3 mM) under aerobic conditions was shown. The WT in static LB without EDTA was used as the control. The relative pellicle formation ((EDTA and indicated cation)/EDTA-absence control) was presented in the figure. EDTA only ('No cation' was used as the negative control. Presented are averages of four replicates with the standard deviation indicated by error bars.

On the basis that metal cations are of general importance in biofilm formation, we examined the effects of certain metal cations on pellicle formation of *S. oneidensis*. The metal chelator ethylenediaminetetraacetate (EDTA) has been shown to have an activity against biofilms of various bacteria by removing metal cations [28,29]. As shown in Figure 3A, 0.3 mM EDTA completely blocked pellicle formation of *S. oneidensis*. A severe inhibitory effect was also observed in the presence of 0.1 and 0.2 mM of EDTA, reducing the pellicles to approximately 50 and 70% (by OD_600 _readings), respectively (Figure 3B). In addition, the pellicle development was much slower than the non-EDTA control. To rule out that the observation was due to toxicity of EDTA to *S. oneidensis*, the same experiment was conducted again under agitated conditions. No noticeable difference in growth between samples containing 0.3 mM EDTA and the non-EDTA control. All these results indicate that EDTA at the tested concentration has a detrimental effect on pellicle formation of *S. oneidensis*.

We reasoned that the inhibitory effect of EDTA on pellicle formation of *S. oneidensis *was due to the absence of free metal cations in the cultures. Therefore, the role of a specific cation in the process can be assessed by the addition of this cation to the cultures containing EDTA. Given that 0.3 mM EDTA appears to be close to the minimal EDTA concentration for complete inhibition of pellicle formation, we chose the concentration for this analysis to determine the importance of a variety of metal cations in pellicle formation. An array of metal cations with different stability constants [log(*K^c^*)] were tested, including Cu(II) [*K^c ^*= 5.77], Mg(II) [*K^c ^*= 8.83], Ca(II) [*K^c ^*= 10.61], Mn(II) [*K^c ^*= 15.6], Zn(II) [*K^c ^*= 17.5], Fe(II) [*K^c ^*= 25.0], and Fe(III) [*K^c ^*= 27.2]. To saturate 0.3 mM EDTA, the concentration for each metal cation used was 0.3 mM as well.

The addition of Ca(II), Mn(II), Cu(II), or Zn(II) fully rescued the initiation of pellicle formation at the cell density threshold and subsequent development (Figure 3A (only Ca(II) was shown), 3C). On the contrary, the inhibitory effect of EDTA was noticeably lessened but not fully removed when Mg(II) was added (Figure 3A). In the case of Fe(II) and Fe(III), the addition of either agent partially rescued (~40%) the pellicle formation defect caused by EDTA (Figure 3A). In addition, unlike pellicles formed in the non-EDTA control or in the presence of Ca(II), Mn(II), Cu(II), or Zn(II), the Fe-enabled pellicles were weakly attached to the container wall and fragile. As a result, the pellicles can be detached from the wall and broken into pieces with a slight shake. The same results were observed with even higher levels of Fe(II) or Fe(III) (up to 0.9 mM). In solution, the addition of an extra amount of certain metal cation may release other cations with lower stability constants from EDTA. However, this is unlikely to be the underlying reason for the observed results because the inhibitory effects of these tested cations on pellicle formation are not correlated to the stability constants of the tested metal cations.

### Progression of pellicle formation was delayed but not prevented in flagella-less *S. oneidensis*

**Figure 4:**
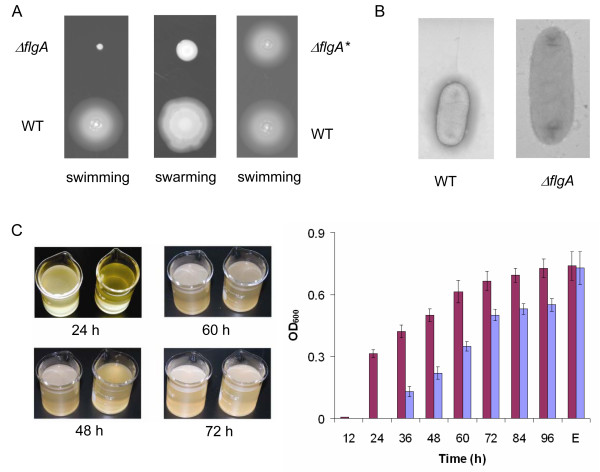
**The Δ*flgA *mutant displayed slow pellicle formation**. (A) Swimming and swarming motility assays of the Δ*flgA *mutant. In both panels, the Δ*flgA *mutant (Upper) was compared to the WT (Lower). The ΔflgA* strain refers to the Δ*flgA *mutant containing pBBR-FLGA. (B) Electron micrographs of WT and the Δ*flgA *mutant. No flagellum was observed on the mutant. (C) Left panel, pellicle formation of the Δ*flgA *mutant. Right panel, the cell densities of cells in pellicles of the WT and the Δ*flgA *mutant. The WT, dark red; the Δ*flgA *mutant, light blue. E represents the time at which the cell density of Δ*flgA *mutant catches up (10 days after inoculation in the experiment). Presented are averages of four replicates with the standard deviation indicated by error bars.

Flagella-less and paralyzed flagellar mutants of many motile bacteria are defective in SSA biofilm and pellicle formation because initial surface attachment depends on flagella-mediated motility [30,31]. However, reports that biofilm and pellicle formation is not affected or even promoted by mutation resulting in impaired flagella in some other bacteria are not scarce [1,32,33]. To assess the role of flagella in pellicle formation of *S. oneidensis*, we tested a flagellum-less strain derived from MR-1 in which *flgA*(*so3253*) was knocked out. FlgA is a molecular chaperone required for P ring assembly in the periplasmic space [34]. The mutant was unable to swarm or swim, indicating that the mutation resulted in functionally impaired flagella (Figure 4A). In addition, the flagella were not found on the mutant under an electron microscope (Figure 4A). To confirm this observation, the intact *flgA *was cloned into plasmid pBBRMCS-5 for complementation. The ability of the mutant to swarm and swim was restored by the corresponding DNA fragment, indicating that the nonmotile phenotype was due to mutation in the gene (Figure 4A).

Compared to MR-1, mutation in *flgA *failed to elicit any significant difference in growth under agitated conditions and SSA biofilm formation (data not shown). However, the mutant displayed a growth defect in the still media and the pellicle formation was drastically delayed. As presented in (Figure 4B), mutation in *flgA *resulted in slow growth with a doubling time of ~7 h, approximately 3 times longer than that of the wild type before pellicles were formed (Figure 1A). Once pellicle formation initiated, that did not occur until 30 h after inoculation, the mutant grew at the rate comparable to the wild type. Interestingly, the development of pellicles in mutants appeared to be normal. As a result, the mutants managed to catch up the wild-type in pellicle production (10 days) (Figure 4B). All of these results suggest that the delayed initiation of pellicle formation of the *flgA *mutant was possibly due to the slow growth of the mutant cells in the unshaken media and flagella were unlikely to play a significant role in the attachment of *S. oneidensis *cells to the wall or pellicle maturation.

### AggA type I secretion pathway is essential in pellicle formation of *S. oneidensis*

Previously, a type I secretion system (TISS) consisting of an ATP-binding protein in the inner membrane RtxB (SO4318), an HlyD-family membrane-fusion protein SO4319, and an agglutination protein AggA (SO4320) was suggested to be important in SSA biofilm formation of *S. oneidensis *[21,22,35]. A following mutational analysis revealed that AggA was critical to hyper-aggregation of the COAG strain, a spontaneous mutant from MR-1 [22]. In the case of SSA biofilm formation, the impact of mutation in *aggA *was rather mild, reducing the robust biofilm-forming capacity of the COAG strain to the level of the wild-type.

**Figure 5:**
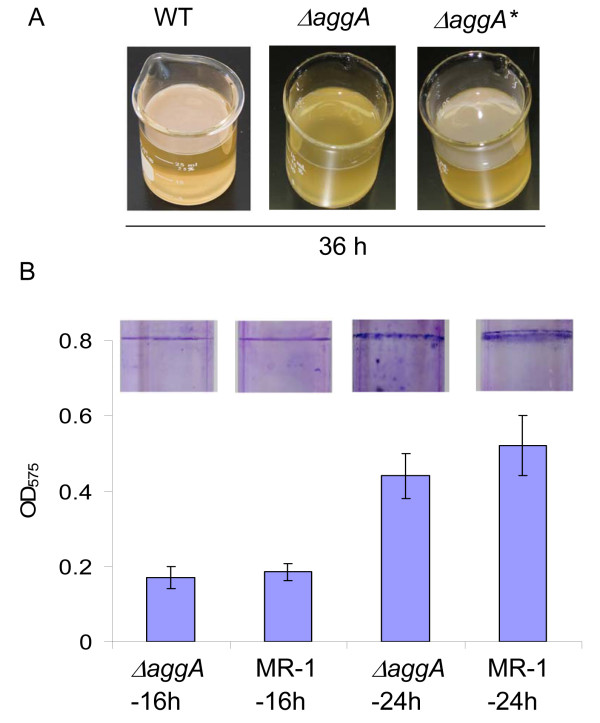
**Biofilm assay of MR-1 and *aggA *mutant**. (A) Pellicle formation of MR-1, Δ*aggA*, ΔaggA* (*aggA *in-frame deletion mutant containing pBBR-AGGA). (B) SSA Biofilm was assessed for the strains indicated after 16 and 24 h, respectively. Cultures were prepared as described in Methods. The averaged OD readings of four independent culture tubes were given with images of representative CV-stained tubes.

Given the importance of AggA in biofilm formation suggested by above-mentioned studies, it is necessary to assess its role in biofilm formation of *S. oneidensis *with a wild-type genetic background. To this end, we constructed an *aggA *in-frame deletion mutant with MR-1 as the parental strain. The physiological characterization revealed that the mutant grew at the rate comparable to that of the parental strain either in the shaking or static conditions. However, the *aggA *mutant was unable to formed pellicles in 5 days (Figure 5A). Introduction of *aggA *on plasmid pBBR-AGGA into the mutant restored its ability to form pellicles, verifying that the phenotype of the *aggA *mutant was specific to the mutation in the *aggA *gene (Figure 5A). As a result, the *aggA *strain displayed a growth pattern different from the wild type strain in the static media by the lack of the growth rate change which signaled the initiation of pellicle formation (Figure 1A). However, the mutant was able to attach to the glass wall at the air-liquid interface, suggesting that AggA is not essential for this step of biofilm formation (Figure 5A). This proposal gained support from the SSA biofilm formation of the mutant, which differed from that of the wild type strain insignificantly (Figure 5B). All these data implicate that AggA TISS is required for pellicle formation, most likely at the monolayer pellicle formation stage, which appears to be different from that in SSA biofilm formation.

## Discussion and Conclusions

In the microbial world, existence within surface-associated structured multicellular communities is the prevailing lifestyle [36,37]. The pellicles of facultative bacteria formed at the liquid-air interface can be selectively advantageous given that respiration with oxygen as the terminal electron acceptor is the most productive. In *S. oneidensis*, the growth rate was promoted by better access to oxygen evidenced by that the cells grew much faster in shaking than in static cultures. Along with the observation that SSA biofilm formation of *S. oneidensis *was inhibited under anaerobic conditions, the requirement of oxygen for pellicle formation may mainly come from its facilitation of aggregation and attachment of cells to the solid surfaces. This is consistent with previous findings that oxygen promotes autoaggregation of and sudden depletion of molecular oxygen was shown to act as the predominant trigger for initiating detachment of individual cells from biofilms [26,38]. We therefore propose that an oxygen gradient established in static cultures with the highest oxygen concentration at the surface resulted in a larger number of cells at the A-L interface to form pellicles, which eventually induce attachment of individual cells to the abiotic surface.

To form pellicles, *S. oneidensis *cultures require certain divalent ions. Involvement of metals in biofilm formation either as a facilitator or an inhibitor has been well documented. In recent years, many elegant studies about the susceptibility of biofilms to metals (as an inhibitor) have been published [39-41]. Although metals as a biofilm formation facilitator have been studied for more than two decades, only a few metals (Ba(II), Mg(II), Ca(II), Fe(III), and Fe(III)) have been investigated [34,42,43]. In *P. aeruginosa*, all these metals but Ba(II) are able to protect *P. aeruginosa *biofilms against EDTA treatment, presumably by stabilizing the biofilm matrix. In addition, it has been shown that there is a positive correlation between calcium concentration and amount of biofilm accumulation [44]. While our data support previous conclusions that calcium plays an important role in stabilizing biofilms of bacteria [34,43,44], most of other findings are either new or surprising. Among tested metal cations, Cu(II), Ca(II), Mn(II), and Zn(II) belong to the same class, which are capable of restoring the ability of *S. oneidensis *to form pellicles in the presence of EDTA completely. In contrast, Mg(II) shows mild effects on relieving EDTA inhibition whereas Fe(II) and Fe(III) counteracted EDTA in a way different from other tested cations evidenced by the fragile pellicles. In combination, these data suggest that the relative stability constants of metal cations (Cu(II) [5.77], Mg(II) [8.83], Ca(II) [10.61], Mn(II) [15.6], Zn(II) [17.5], Fe(II) [25.0], and Fe(III) [27.2]) and their affect on EDTA inhibition are not correlated.

It is particularly worth discussing roles of Fe(II) and Fe(III) in pellicle formation of *S. oneidensis*. In recent years, many reports have demonstrated that the iron cations are important, if not essential, in bacterial biofilm formation [34,45-47]. In *P. aeruginosa*, influence of Fe(II) and Fe(III) on the process was equivalent to that of Ca(II) [34]. In *S. oneidensis*, irons in forms of Fe(II) and Fe(III) were not only unable to neutralize the inhibitory effect of EDTA on pellicle formation completely but also resulted in structurally impaired pellicles although these agents indeed play a role in pellicle formation. This observation indicates that irons are not so crucial as Cu(II), Ca(II), Mn(II), and Zn(II) in pellicle formation of *S. oneidensis*. In fact, this may not be surprising. In *Acinetobacter baumannii *and *Staphylococcus aureus*, iron limitation improved biofilm formation [48,49]. Therefore, it is possible that different bacteria respond to irons in a different way with respect to biofilm formation.

Like SSA biofilms, pellicles require EPS to form a matrix to support embedded cells. Although EPS are now widely recognized as the essential components for biofilm formation and development in all biofilm-forming microorganisms studied so far, diversity in their individual composition and relative abundance of certain elements is substantial [50]. For example, extracellular nucleic acids, which are not important in most biofilm-forming microorganisms, are required for SSA biofilm formation in a variety of bacteria [11,36,37,51,52]. In *S. oneidensis*, proteins not extracellular DNAs are required to pellicle formation. While essential extracellular proteins for *S. oneidensis *pellicle formation are largely unknown, results from this study demonstrated that the AggA TISS is crucial in the process, likely at the development of the monolayer. One of substrates of this transporter is predicted to be SO4317, a large 'putative RTX toxin' [35], implicating that the protein may be involved in pellicle formation. In the case of polysaccharides, mannose dominates not only in pellicles but also in supernatants, implicating that mannose-based polysaccharides may have a more general role in the bacterial physiology.

Like in *B. subtilis*, mutations in *S. oneidensis *flagellar genes resulting in the nonmotile phenotype significantly delayed the initiation and development of pellicle formation [17]. Here we further illustrated that neither SSA biofilm formation nor the maturization of pellicle was impaired by the mutations. In agreement with findings on biofilm formation of *Bacillus cereus *[13], this observation suggests that motility not only promotes cells to move to surfaces where the pellicle forms but also facilitate planktonic cells entrance into the pellicle.

Overall, the results presented here provided the first insights into pellicle formation of *S. oneidensis*, making pellicle formation of *S. oneidensis *a simple research model for biofilm formation in general. The study highlights parallels and significant differences between this process and well-documented paradigms, raising some key questions demanding immediate investigations. These include what the major polysaccharides in *S. oneidensis *pellicles are, why irons result in fragile pellicles in the presence of EDTA, and which proteins and their secretion pathway(s) are directly related to pellicle formation.

## Methods

### Bacterial strains, plasmids, and culture conditions

Bacterial strains and plasmids used in this study are listed in Table 1[53]. *Escherichia coli *and *S. oneidensis *strains were routinely grown in LB broth or on LB plates at 37°C and the room temperature for genetic manipulation, respectively. When needed, antibiotics were used at the following concentrations: ampicillin at 50 μg/ml and gentamycin at 15 μg/ml.

**Table 1:** Strains and plasmids used in this study

Strain or plasmid	Relevant genotype	Reference or source
*E. coli*		
WM3064	Donor strain for conjugation; *ΔdapA*	[53]
*S. oneidensis*		
MR-1	Wild-type	ATCC 700550
JZ3253	*flgA *deletion mutant derived from MR-1; *Δ flgA*	This study
JZ4320	*aggA *deletion mutant derived from MR-1; *ΔaggA*	This study
		
Plasmid		
pDS3.0	Ap^r^, Gm^r^, derivative from suicide vector pCVD442	Lab stock
pBBR1MCS-5	Gm^r ^vector used for complementation	Lab Stock
pDS-AGGA	*aggA *deletion construct in pDS3.0	This study
pDS-FLGA	*flgA *deletion construct in pDS3.0	This study
pBBR-AGGA	pBBR1MCS-5 containing *aggA *of *S. oneidensis*	This study
pBBR-FLGA	pBBR1MCS-5 containing *flgA *of *S. oneidensis*	This study

### Pellicle formation, measurement of growth, and quantification of pellicles

A fresh colony grown overnight on a LB plate was used to inoculate 50 ml LB and incubated in a shaker (200 rpm) to an OD_600 _of 0.8 at the room temperature. This culture was then diluted 500-fold with fresh LB, resulting in the starting cultures. Throughout the study, all starting cultures of *S. oneidensis *strains were prepared this way. Aliquots of 30 ml starting cultures were transferred to 50 ml Pyrex beakers. The beakers were kept still for pellicle formation at the room temperature and dissolved oxygen (DO) of the cultures was recorded every hour with an Accumet XL40 meter (Fisher Scientific). M1 defined medium containing 0.02% (w/v) of vitamin-free Casamino Acids and 15 mM lactate with one of electron acceptors including fumarate (20 mM), nitrate (5 mM), trimethylamine *N*-oxide (TMAO) (20 mM), dimethyl sulfoxide (DMSO) (20 mM) and ferrous citrate (10 mM), was used to test pellicle formation in the defined medium [54]. To separate cells in pellicle and underneath, cultures were withdrawn carefully for collecting planktonic cells and the left pellicles. For growth measurement, 27 parallel starting cultures were used and 3 were collected at each time point and the rest remained undisturbed. The cell density (OD_600_) of cultures containing planktonic cells was measured first as the planktonic cell density and measured again as the overall cell density after cells from pellicles were added and extensively vortexed. To quantify the pellicles formed by the *S. oneidensis *wild-type and mutant strains, cells from pellicles were collected, suspended in 30 ml fresh LB, violently vortexed, and applied to the spectrometer at 600 nm.

### Proteinase K and DNase I treatment of *S. oneidensis *pellicles

*S. oneidensis *was statically cultured in LB broth with the addition of proteinase K (0 μg/mL, 100 μg/mL, and 500 μg/mL) or DNase I (Qiagen, 0U/mL, 100U/mL, 500U/mL and 1000U/mL) for 3 days [55]. We also investigated whether these 3 enzymes could dissolve established pellicles. 2-day old pellicles were rinsed with 20 mM Tris-HCl (pH = 8.0) and incubated in the same buffer supplemented with proteinase K at 37°C for 2 days. Similarly, 2-day old pellicles were incubated with DNase I to examine the DNA content at room temperature for 2 days.

### Mutagenesis, physiological characterization and complementation of the resulting mutants

Deletion mutation strains were constructed using the fusion PCR method illustrated previously [56]. Primers used for mutagenesis were listed in Additional file 1. In brief, two DNA fragments flanking the target gene were generated from *S. oneidensis *genomic DNA by PCR with primers 5F/5R and 3F/3R, respectively. Fusion PCR was then performed to join these two DNA fragments with primers 5F/3R. The resulting single fragment was digested with *Sac*I and ligated into the *Sac*I-digested and phosphatase-treated suicide vector pDS3.0. The resultant vectors were electroporated into the donor strain, *E. coli *WM3064 and then moved to *S. oneidensis *by conjugation. Integration of the mutagenesis construct into the chromosome and resolution were performed to generate the final deletion strains. The deletion was verified by PCR and DNA sequencing.

For complementation, DNA fragments containing *aggA *or *flgA *were generated by PCR amplification with MR-1 genomic DNA as the template using primers SO4320-COM-F/SO3988-COM-R and SO3253-COM-F/SO3253-COM-R, respectively as listed in Additional file 1. These fragments were digested with *Sac*I and ligated to *Sac*I-digested pBBR1MCS-5 to form pBBR-AGGA and pBBR-FLGA, which was electroporated into WM3064. Introduction of pBBR-AGGA or pBBR-FLGA into the corresponding mutant was done by conjugation, and gentamycin-resistant colonies were selected. The presence of pBBR-AGGA or pBBR-FLGA in the corresponding mutant was confirmed by plasmid purification and restriction enzyme digestion.

### Swarm and swimming motility assay

A fresh colony of tested strains was grown to an OD_600 _of 0.8 in LB media. The cultures (1 ml) were spotted onto a swarm LB plate (0.5% agar) or stabbed into a swimming LB plate (0.2% agar). All plates were incubated at the room temperature for 48 h. Images were acquired using Alpha Innotech's Fluorchem imaging system.

### SSA biofilm assay

The SSA biofilm formation assay used is based on the method previously reported [57]. In brief, 3 ml of fresh LB in 15 ml glass tubes were inoculated with *S. oneidensis *strains from an overnight culture in LB at 200 rpm. After 16, 24, 32, or 40 h of incubation at 200 rpm at room temperature, 500 μl of 1% (wt/vol) crystal violet (CV) solution was added to each tube and incubated for 15 min. Tubes were rinsed three times with 5 ml of distilled H_2_O and air dried. Biofilm formation was quantified by measuring the absorbance at 575 nm. Each assay was performed four times.

### Thin layer chromatography (TLC) analysis

Supernatants and pellicles were collected after 36 h of growth in static LB media. Pellicles were treated with 100 μg/mL proteinase K for removal of cells. Cell-less pellicles and supernatants were subjected to exopolysaccharide extraction and hydrolysis with trifluoroacetic acid as described previously [58]. The resulting monosaccharides were dissolved in ddH_2_O in the concentration of 10 mg/ml, and 2 μl of the sample was spotted onto TLC plates (silica gel 60 F_254_; Merck). After development in butan-1-ol-acetone-water (4:5:1), the TLC plates were dipped in the reagent aniline-diphenylamine in acetone and incubated for 2 to 5 min at 100°C.

## Authors' contributions

YL carried out pellicle formation and characterization experiments and drafted the manuscript. HG conceived of the study, and participated in its design, and directed all experiments and coordination and drafted the manuscript. JC carried out the mutagenesis experiments. YD and LW carried out SSA biofilm and TLC assays. ZH participated in design of the study and helped to draft the manuscript. XL and GQ participated in the design of the study and helped to draft the manuscript. JZ conceived of the study, and participated in its design and coordination and helped to draft the manuscript. All authors read and approved the final manuscript.

## Supplementary Material

Additional file 1 **Primers used in this study**. File contains all primers used in this study Click here for file
