# Supplementary material for: Pellicle formation in Shewanella oneidensis
Source: BMC Microbiol. 2010 Nov 16;10:291. doi: 10.1186/1471-2180-10-291 (PMC2995470; doi:10.1186/1471-2180-10-291)
Supplement: Additional file 1 — Primers used in this study. File contains all primers used in this study [file 1471-2180-10-291-S1.PDF]

Additional file 1. Primers used in this study

Mutagenesis	
SO3253-5-F	GGGAGCTCTAGGCAATGTCGTTAACGGT
SO3253-5-R	GCTCCTCGACAATAATAGCACTTTTATCGGCGACGCGCAT
SO3253-3-F	TGCTATTATTGTCGAGGAGCGTAATATTGGCGACCAAATC
SO3253-3-R	AAGAGCTCCTACCTGAGATGATTGCTCG
SO4320-5-F	GGGAGCTCAGGATAAGGTAAGTAAAGC
SO4320-5-R	GCTCCTCGACAATAATAGCAGGCTTTGAGCGGATAGTG
SO4320-3-F	TGCTATTATTGTCGAGGAGCCAACGCAGCTTACTCGA
SO4320-3-R	AAGAGCTCGGACGACTTGAACCATAGC
Complementation	
SO3253-COM-F	GGGAGCTCCTTCTGACACCAGATCTCCTAG
SO3253-COM-R	AAGAGCTCGGCTCCAGCGTCATGTATAC
SO4320-COM-F	GGGAGCTCCATCACCGGACAAAGATCC
SO4320-COM-R	AAGAGCTCCGTCACGCTAAGTAATAGG
